# Development, Validation, and Reliability Analysis of the Household-Related Air Pollution on the Childhood Bronchial Asthma Onset Checklist (HAPBAC-Checklist)

**DOI:** 10.7759/cureus.70658

**Published:** 2024-10-01

**Authors:** Muhammad Naim Ibrahim, Nik Rosmawati Nik Husain, Mohamad Ikram Ilias, Kueh Y Cheng, Norzaihan Hassan, Nurul Ainun Hamzah, Norazlin Idris, Khairul Azuan Che Azid

**Affiliations:** 1 Community Medicine Department, School of Medical Sciences, Health Campus, Universiti Sains Malaysia, Kota Bharu, MYS; 2 Pediatrics Department, School of Medical Sciences, Health Campus, Universiti Sains Malaysia, Kota Bharu, MYS; 3 Biostatistics and Research Methodology Unit, School of Medical Sciences, Health Campus, Universiti Sains Malaysia, Kota Bharu, MYS; 4 Kota Bharu Health Clinic, Kota Bharu District Health Office, Kota Bharu, MYS; 5 Environmental and Occupational Health Department, School of Health Sciences, Health Campus, Universiti Sains Malaysia, Kota Bharu, MYS; 6 Occupational and Environmental Health Laboratory, School of Health Sciences, Health Campus, Universiti Sains Malaysia, Kota Bharu, MYS

**Keywords:** air pollution, checklist, childhood asthma, household, indoor air quality

## Abstract

Introduction

Most studies on indoor air pollution for childhood bronchial asthma (BA) were focused on institutions such as schools. However, many asthma triggers are household sources such as consumer products, smoking, and pets. In Malaysia, there is no specific checklist to evaluate the risk of childhood BA onset from household factors. Thus, the study aims to develop and validate an observation checklist for assessing household-related air pollution on childhood BA onset.

Methodology

The study was conducted in Kota Bharu Kelantan from March to November 2023. The development of the checklist was conducted in five stages: 1) the construction of domains and items from the existing literature, 2) interviews with the parents, 3) content validation by ten experts (item-level content validity index (I-CVI) and scale-level content validity index (S-CVI), 4) face validation by 12 experts (item-level face validity index (I-FVI) and scale-level face validity index (S-FVI)), and (5) reliability analysis (kappa agreement analysis) involving 20 houses assessed by two assessors.

Results

The initial draft of the checklist contained five domains with 57 items: sociodemographic (19 items), family history (three items), child’s medical history (six items), household attributes (19 items), and outdoor attributes (10 items). The I-CVI scores ranged from 0.90 to 1.00, indicating good relevancy. The S-CVI value was 0.97, showing a satisfactory level. The I-FVI was at least 0.96, and the S-FVI of 0.98 indicating the participants easily understood the checklist. The kappa analysis for five domains combined was 0.92 (95%CI: 0.89-0.95). The final validated checklist consists of five domains with 59 items.

Discussion and conclusion

The Household-Related Air Pollution on the Childhood Bronchial Asthma Onset Checklist (HAPBAC-Checklist) was designed to address household air pollution originating from indoor sources. However, recognizing the impact of outdoor factors through various pathways, along with considering family history of atopy and medical conditions, is important. The checklist, developed in Malay, aims to enhance utilization and adoption, particularly among local healthcare professionals and authorities. The checklist is a valid and reliable tool for assessing household-related air pollution on childhood BA onset. This novel checklist significantly benefits screening indoor air quality, crucial for preventing childhood BA.

## Introduction

Asthma affects more than 300 million people worldwide and is the most common chronic disease responsible for significant morbidity and mortality among children [1]. Between 2019 and 2019, its global incidence increased by 13.0%-37 million cases. Notably, children exhibit a higher prevalence of bronchial asthma (BA) at 9.1%, while adults experience BA at 6.6% [2]. The prevalence of childhood BA in Malaysia exhibited a striking 50.0% increase, rising from 4.7% in 1996 to 7.1% in 2011 [3]. This trend persists in its upward trajectory globally.

The disease is heterogeneous, that is, driven by genetic and environmental interaction. Studies among twins have shown that genetic factors explained 24.0%-34.0% of the variation in childhood BA onset, while environmental factors accounted for much more, from 66.0% to 76.0% [4,5]. Gene heritability has remained constant worldwide, but childhood BA incidence has increased several-fold over the last 100 years. The situation strongly correlates with environmental factors, with urbanization and industrialization, resulting in increased pollutants and allergens and driving climate and biodiversity changes [6].

Childhood BA prevalence is higher in developed countries. For instance, the prevalence was 15.9% in the United States, 12.1% in Japan, 11.9% in the United Arab Emirates, 11.1% in Australia, 10.1% in the United Kingdom, 10.0% in Spain, and 9.1% in Singapore. On the other hand, developing countries tended to have lower prevalence: Malaysia reported 7.1%, Indonesia 6.7%, Mexico 6.2%, and Vietnam 6.0% [7,8]. In addition, urban residents were reported to have a higher prevalence of childhood BA; in Malaysia, Kuala Lumpur had a 13.9% rate, compared to 6.3% in Muar [3].

According to Habre et al., young children in urban areas spend 80.0%-90.0% of their time at home, indicating a higher percentage of exposure to a broad portfolio of household air pollutants [9]. The growing popularity of indoor games and video games and parental concerns about outdoor hazards and dangers have led to changes in children’s lifestyles [10]. Consequently, household air quality plays a crucial role in the well-being and performance of children.

Most common household air pollutants are associated with occupancy activities such as cooking, cleaning, smoking, pets, and indoor combustion. Studies in different countries, including Malaysia, have found that household air pollution levels were up to five times higher than outdoor air, due largely to poor ventilation and various household attributes [11-13]. Outdoor sources can also affect household air quality and cannot be neglected when studying household air pollution [14]. Residents in proximity to heavy traffic or industrial and farming activities, for example, are at risk of poor household air quality [15].

However, awareness of the risk of childhood BA onset due to household air pollution in developing countries, including Malaysia, remains limited, which complicates mitigation efforts. Most studies on indoor air pollution for childhood BA onset in Malaysia were conducted at institutions such as preschools and in school environments, but they represent only 27.0% of children’s daily schedules [16]. In addition, the absence of locally tailored household checklists for assessing the risk of childhood BA onset poses a challenge because of the home’s distinctive sociodemographic and cultural context. Most of the checklists available were designed for indoor spaces in general rather than specifically for household assessment [17].

Therefore, the present study aims to develop and validate the Household-Related Air Pollution on Childhood BA Onset Checklist (HAPBAC-Checklist) for assessing the risk of childhood BA in Malaysian households. The checklist is intended to be used by researchers and trained personnel to record visual observations from the household environment and interview parents or guardians in Malaysian households.

## Materials and methods

The study was conducted in Kota Bharu, Kelantan, Malaysia, from March to November 2023. The checklist was developed in the Malay language, and the procedure involved four stages.

Stage 1: Development of the HAPBAC-Checklist

**Figure 1:**
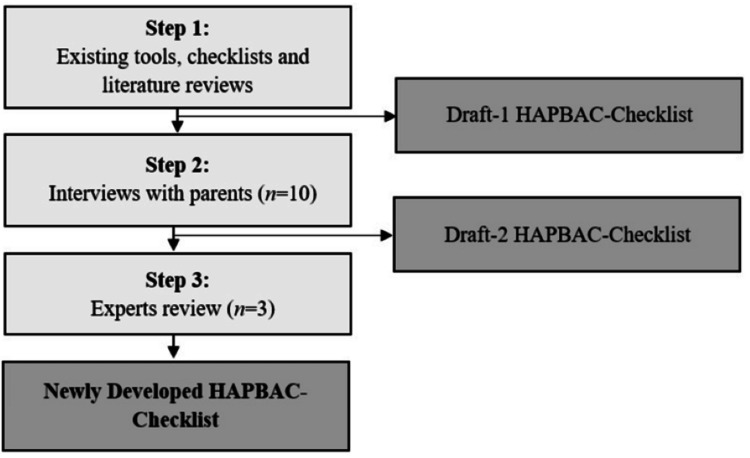
Flowchart for the development of the HAPBAC-Checklist

The first stage began by selecting the related domains and items based on the three-step process adapted from Curl et al. [18]: i) existing tools, checklists, and literature reviews; ii) interviews with parents; and iii) expert reviews (see Figure 1). A final draft of the newly developed HAPBAC-Checklist was constructed after completing all three stages.

Step 1: Existing Tools, Checklists, and Literature Reviews

An extensive review of existing tools and checklists was conducted to choose the appropriate domains and items for childhood BA onset risk and household air pollution. The home characteristics and asthma triggers on the Checklist for Home Visitors by the U.S. Environmental Protection Association (2018) were used as a starting point [19]. The checklist contains recent and relevant household-sourced allergens relevant to childhood BA. Next, further pertinent checklists by the International Laboratory for Air Quality and Health (2017) and Syazwan et al. were reviewed [20,21]. Then, extensive literature reviews were conducted on the relevant current household determinants for childhood BA onset. Finally, significant determinants were identified, and the initial item pool was generated for the Draft-1 HAPBAC-Checklist.

Step 2: Interviews with Parents

Parents’ perspectives on and experiences with childhood BA determinants were gained to refine the checklist, as recommended by Guest et al. [22]. The interviews were conducted with 10 parents of children living in Kota Bharu and newly diagnosed with BA at the Hospital Universiti Sains Malaysia (HUSM) from 2021 to 2023. According to Guest et al., 10 respondents is an optimal number because 92% of concepts can be identified in the process of checklist development. The parents were recruited using convenience sampling during their children’s visits to the pediatric clinic at the HUSM and were conducted in a one-on-one format. The parents were interviewed using the Draft-1 HAPBAC-Checklist and encouraged to comment on possible ways to improve the checklist items regarding childhood BA risk factors. Additional relevant findings were used to refine the checklist into the Draft-2 HAPBAC-Checklist.

Step 3: Expert Review

Three experts were selected from public health physicians, pediatricians, and family medicine physicians (FMS) from HUSM, HRPZII, and PKD KB. Deep discussions on the determinants of childhood BA and household air pollution were held in multiple one-on-one sessions to improve the checklist. At this stage, a refined checklist was generated and called the Newly Developed HAPBAC-Checklist.

Stage 2: Content validation

The content validation of the checklist was performed by a panel of six experts: two public health physicians, two health environmentalists, one pediatrician, and one family medicine physician. The numbers on the expert panel followed previous studies’ recommendations [23,24]. The expert panel was handed the checklist with information regarding its development, evaluation guidelines, and analysis requirements. The panel was asked to analyze and clarify the importance of each domain and item by providing a score for each item. The evaluation score on the constructed items was based on the level of relevance using a four-point scale: 0 (not relevant), 1 (somewhat relevant), 2 (quite relevant), and 3 (highly relevant) [24]. The experts were encouraged to provide written comments where necessary to improve the relevance of the items. Finally, the results were measured using the item-level content validity index (I-CVI) and the scale-level content validity index (S-CVI), with a value of at least 0.83 deemed acceptable [24]. The panel’s comments were also described. At this stage, the Draft-3 HAPBAC-Checklist was generated.

Stage 3: Face validation

Next, face validity was assessed by 12 raters, as suggested [25,26]. The raters were two public health physicians, two medical officers, two paramedics (one nurse and one medical assistant), four health and environmental inspectors, and two undergraduates in environmental studies. The raters were chosen based on their direct involvement in the health environmental evaluation and proficiency in the Malay language. The raters evaluated the clarity and comprehensibility of instruction and language used in the Draft-3 HAPBAC-Checklist. The evaluation score on the constructed items was based on the level of clarity and comprehensibility, using a four-point scale: 0 (not clear), 1 (somewhat clear), 2 (quite clear), and 3 (very clear) [26]. The experts were encouraged to provide written comments where necessary to improve the clarity and comprehension of the items. Finally, the results were measured using the item-level face validity index (I-FVI) and the scale-level face validity index (S-FVI), with an acceptable value of at least 0.83 [26]. The raters’ comments were also described. At this stage, the Draft-4 HAPBAC-Checklist was generated.

Stage 4: Reliability analysis

Reliability was assessed by inter-rater reliability analysis with the kappa agreement in view of most items in the checklist with dichotomous (yes/no) answers. The kappa agreement is a reliability index that shows the degree of agreement on consistency on dichotomous classification between raters using the checklist [27]. The two raters were a health environmental inspector and a community nurse who performed data collection at 20 children’s homes, as recommended by Blackman et al. [28]. The raters used the same checklist, the Draft-4 HAPBAC-Checklist, and conducted their work simultaneously, independently, and without conferring during the evaluation. The completed checklists were collected at the end of the observation. The kappa inter-rater reliability was analyzed using Statistical Product and Service Solutions (SPSS, version 26.0; IBM SPSS Statistics for Windows, Armonk, NY) to calculate Cohen’s kappa value, κ. The recommended and acceptable value of κ for reliability is at least 0.80 (80.0%) [29]. At this stage, the validated HAPBAC-Checklist was generated.

## Results

Development of the HAPBAC-Checklist

The Draft-1 HAPBAC-Checklist produced from step i) - existing tools, checklists, and literature reviews - consisted of five domains and 57 items, as shown in Table 1.

**Table 1:** Items in Draft-1 HAPBAC-Checklist (Step 1)

Domain	Number of items	Concept measured	Response option
Sociodemographic	19	Socioeconomic, cultural and living background	Numerical and single or multiple choice
Family history	3	Family history of BA	Numerical and single choice
Medical history	6	Risk factors for developing childhood BA	Single choice
Household attributes	19	Household pollution source	Single choice
Outdoor attributes	10	Outdoor pollution source	Single choice

Feedback from parents on the Draft-1 HAPBAC-Checklist comprised step 2. Among the 10 parents interviewed, 6 (60%) were mothers, and four (40%) were fathers. Their valuable insights were instrumental in refining the checklist, resulting in the construction of the Draft-2 HAPBAC-Checklist. In Step 3, three experts - a public health physician, a pediatrician and a family medicine physician - reviewed the Draft-2 HAPBAC-Checklist. The checklist was refined, and the Newly Developed HAPBAC-Checklist was generated. The review findings are shown in Table 2.

**Table 2:** Experts' review of the Newly Developed HAPBAC-Checklist (Step 3)

Domain	Number of Items change	Number of items	Description for changes
Sociodemographic	0	19	All items remained the same. Refinements on numbering and sentences were made.
Family history	2	3	One item on parents’ history of asthma was separated into two items: father and mother. One item on the number of siblings was removed because it was irrelevant.
Medical history	0	6	All items remained the same. Sentence refinements were made.
Household attributes	0	19	All items remained the same. Sentence refinements were made.
Outdoor attributes	0	11	All items remained the same. Sentence refinements were made.

Content validation

A panel of six experts conducted the content validation process for the Newly Developed HAPBAC Checklist. The process consisted of two rounds. In the first round, the I-CVI scores for each item ranged from 0.50 to 1.00, while the S-CVI scores for each domain ranged from 0.89 to 1.00, with the global S-CVI/Ave at 0.94. Improvement was made by removing three items with I-CVI scores below 0.83 and adding five items relevant to childhood BA risk, as recommended by the panel.

As a result, the Draft-3 HAPBAC Checklist featuring 59 items was generated. The I-CVI scores for each item ranged from 0.83 to 1.00. The S-CVI scores for each domain ranged from 0.90 to 1.00, with a global S-CVI/Ave improvement of 0.97. The final findings are shown in Table 3. The content validation results indicate good relevance of the items constructed in the five domains.

**Table 3:** Content validity index for five domains based on the relevancy rating of items by six experts ^a^I-CVI: Item-Level Content Validity Index ^b^S-CVI/Ave: Scale-Level Content Validity Index Based on the Average Method

Items	Experts						Expert in agreement	^a^I-CVI
	1	2	3	4	5	6		
Domain 1: Sociodemographic								
Age	1	1	1	1	1	1	6	1.00
Sex	1	1	1	1	1	1	6	1.00
Race	1	1	1	1	1	1	6	1.00
School location/name	1	1	1	1	0	1	5	0.83
School session	1	1	1	1	1	1	6	1.00
Other additional school/care centre	1	1	1	1	1	1	6	1.00
Occupation of father	1	1	1	0	1	1	5	0.83
Occupation of mother	1	1	1	0	1	1	5	0.83
Highest education of father	1	1	0	1	1	1	5	0.83
Highest education of mother	1	1	1	1	1	1	5	0.83
Household income per month	1	1	1	1	1	1	5	0.83
House location	1	1	1	1	1	1	6	1.00
Duration (years) of stay at current house	1	1	1	1	1	1	6	1.00
House type	1	1	1	1	1	1	6	1.00
House structure type	1	1	1	1	1	1	6	1.00
Number of bedrooms	1	1	1	1	1	1	6	1.00
Number of households	1	1	1	1	1	1	6	1.00
							^b^S-CVI/Ave	0.94
Domain 2: Family History								
Father’s atopy history	1	1	1	1	1	1	6	1.00
Mother’s atopy history	1	1	1	1	1	1	6	1.00
Sibling’s atopy history	1	1	1	1	1	1	6	1.00
							^b^S-CVI/Ave	1.00
Domain 3: Medical history								
Gestation at birth	1	1	1	1	1	1	6	1.00
History of mother smoking during pregnancy	1	1	1	0	1	1	5	0.83
Exclusive breastfeeding	1	1	1	0	1	1	5	0.83
Congenital illness	1	1	0	1	1	1	5	0.83
Ward admission or lung infection	1	1	1	1	1	1	6	1.00
Other illness	1	1	1	1	1	1	6	1.00
Prolonged use of prescribed medication	0	1	1	1	1	1	5	0.83
							^b^S-CVI/Ave	0.90
Domain 4: Household attributes								
Parents smoking	1	1	1	1	1	1	6	1.00
Any household smoke indoor	1	1	1	1	1	1	6	1.00
Any household vape indoor	1	1	1	1	1	1	6	1.00
Presence cigarette but or ashtray	1	1	1	1	1	1	6	1.00
Pet indoor	1	1	1	1	1	1	6	1.00
Carpet or fabric furniture at living room	1	1	1	1	1	1	6	1.00
Presence of mold	1	1	1	1	1	1	6	1.00
Use of aerosol insecticide	1	1	1	1	1	1	6	1.00
Use of mosquito coil	1	1	1	1	1	1	6	1.00
Herbicide/insecticide storage	1	1	1	1	1	1	6	1.00
New wood furniture	1	1	0	1	1	1	5	0.83
Newly painted house structure	1	1	1	1	1	1	6	1.00
Air conditioner use	1	1	1	1	1	1	6	1.00
Frequency house cleaning	1	1	1	1	1	1	6	1.00
Main cleaning method	1	1	1	1	1	1	6	1.00
Aerosol cleaning product use	1	1	1	1	1	1	6	1.00
Window at kitchen	1	1	1	1	1	1	6	1.00
Exhaust fan at kitchen	1	1	1	1	1	1	6	1.00
Wooden stoves indoor	1	1	1	1	1	1	6	1.00
Perfume indoor	1	1	1	1	1	1	6	1.00
HEPA air filter use	1	1	1	1	1	1	6	1.00
							^b^S-CVI/Ave	0.99
Domain 5: Outdoor attributes								
Car parking at connected house balcony	1	1	1	1	1	1	6	1.00
Motorcycle parking nearby	1	1	0	1	1	1	5	0.83
Wood stoves outdoor nearby	1	1	1	1	1	1	6	1.00
Domestic garbage burning nearby	1	1	1	1	1	1	6	1.00
Main road nearby	1	1	1	1	1	1	6	1.00
Municipal landfill <500m	1	1	1	1	1	1	6	1.00
Industrial area <500m	1	1	1	1	1	1	6	1.00
Agricultural area <500m	1	1	1	1	1	1	6	1.00
Mines or quarry <500m	1	1	1	1	1	1	6	1.00
Construction site <500m	1	1	1	1	1	1	6	1.00
Outdoor poultry or pet	1	1	1	1	1	1	6	0.83
							^b^S-CVI/Ave	0.98

Face validation

Twelve raters, consisting of two public health physicians, two medical officers, two paramedics, four health and environmental inspectors, and two undergraduates in environmental studies, assessed the face validity of the Draft-3 HAPBAC Checklist. The I-FVI value for each item ranged from 0.83 to 1.00, and the S-FVI scores for each domain ranged from 0.96 to 1.00, with a global S-FVI/Ave of 0.98. The final findings are shown in Table 4. The results indicated good clarity and comprehensibility, and the Draft-4 HAPBAC-Checklist was generated.

**Table 4:** Face validity index for five domains based on the clarity and comprehensibility rating of items by 12 raters ^a^I-FVI: Item-Level Face Validity Index ^b^S-FVI/Ave: Scale-Level Face Validity Index Based on the Average Method ^c^HEPA: High-Efficiency Particulate Air (filter)

Items		Experts											Rater in agreement	^a^I-FVI
	1	2	3	4	5	6	7	8	9	10	11	12		
Domain 1: Sociodemographic														
Age	1	1	1	1	1	1	1	1	1	1	1	1	12	1.00
Gender	1	1	1	1	1	1	1	1	1	1	1	1	12	1.00
Race	1	1	1	1	1	1	1	1	1	1	1	1	12	1.00
School location/name	1	1	1	0	1	1	1	1	0	1	1	1	10	0.83
School session	1	1	1	1	1	1	1	1	1	1	1	1	12	1.00
Other additional school/care centre	1	1	1	1	1	1	1	1	1	1	1	1	12	1.00
Occupation of father	1	1	1	1	1	1	1	1	1	1	1	1	12	1.00
Occupation of mother	1	1	1	1	1	1	1	1	1	1	1	1	12	1.00
Highest education of father	1	1	0	1	1	1	1	1	1	0	1	1	10	0.83
Highest education of mother	1	1	0	1	1	1	1	1	1	0	1	1	10	0.83
Household income per month	1	1	1	0	1	1	1	1	1	1	1	1	11	0.92
House location	1	1	1	1	1	1	1	1	1	1	0	1	11	0.92
Duration (years) of stay at current house	1	1	1	1	1	1	1	1	1	1	1	1	12	1.00
House type	1	1	1	1	1	1	1	1	1	1	1	1	12	1.00
House structure type	1	1	1	1	1	1	1	1	1	1	1	1	12	1.00
Number of bedrooms	1	1	1	1	1	1	1	1	1	1	1	1	12	1.00
Number of households	1	1	1	1	1	1	1	1	1	1	1	1	12	1.00
													^b^S-FVI/Ave	0.96
Domain 2: Family history														
Father’s atopy history	1	1	1	1	1	1	1	1	1	1	1	1	12	1.00
Mother’s atopy history	1	1	1	1	1	1	1	1	1	1	1	1	12	1.00
Sibling’s atopy history	1	1	1	1	1	1	1	1	1	1	1	1	12	1.00
													^b^S-FVI/Ave	1.00
Domain 3: Medical history														
Gestation at birth	1	1	1	1	1	1	1	1	1	1	1	1	12	1.00
History of mother smoking during pregnancy	1	1	1	1	1	1	1	1	1	1	1	1	12	1.00
Exclusive breastfeeding	1	1	1	1	1	1	1	1	1	1	1	1	12	1.00
Congenital illness	1	0	0	1	1	1	1	1	1	1	1	1	10	0.83
Ward admission or lung infection	1	1	1	1	1	1	1	1	1	1	1	1	12	1.00
Other illness	1	1	1	1	1	1	1	1	1	1	1	1	12	1.00
Prolonged use of prescribed medication	1	1	1	1	1	1	1	1	1	1	1	1	12	1.00
													^b^S-FVI/Ave	0.98
Domain 4: Household attributes														
Parents smoking	1	1	1	1	1	1	1	1	1	1	1	1	12	1.00
Any household smoking indoor	1	1	1	1	1	1	1	1	1	1	1	1	12	1.00
Any household vape indoor	1	1	1	1	1	1	1	1	1	1	1	1	12	1.00
Presence of cigarette but or ashtray	1	1	1	1	1	1	1	1	1	1	1	1	11	0.92
Pet indoor	1	1	1	1	1	1	1	1	1	1	1	1	12	1.00
Carpet or fabric furniture in the living room	1	1	1	1	1	1	1	1	1	1	1	1	12	1.00
Presence of mold	1	1	1	1	1	1	1	1	1	1	1	1	12	1.00
Use of aerosol insecticide	1	1	1	1	1	1	1	1	1	1	1	1	12	1.00
Use of mosquito coil	1	1	1	1	1	1	1	1	1	1	1	1	12	1.00
Herbicide/insecticide storage	1	1	1	1	1	1	1	1	1	1	1	1	12	1.00
New wood furniture	1	1	1	1	1	1	1	1	1	1	1	1	12	1.00
Newly painted house structure	1	1	1	1	1	1	1	1	1	1	1	1	12	1.00
Air conditioner use	1	1	1	1	1	1	1	1	1	1	1	1	12	1.00
Frequency of house cleaning	1	1	1	1	1	1	1	1	1	1	1	1	12	1.00
Main cleaning method	1	1	1	1	1	1	1	1	1	1	1	1	12	1.00
Aerosol cleaning product use	1	1	1	1	1	1	1	1	1	1	1	1	12	1.00
Window in kitchen	1	1	1	1	1	1	1	1	1	1	1	1	12	1.00
Exhaust fan in kitchen	1	1	1	1	1	1	1	1	1	1	1	1	12	1.00
Wooden stoves indoor	1	1	1	1	1	1	1	1	1	1	1	1	12	1.00
Perfume indoor	1	1	1	1	1	1	1	1	1	1	1	1	10	0.83
^c^HEPA filter use	1	1	1	1	1	1	1	1	1	1	1	1	12	1.00
													^b^S-FVI/Ave	0.99
Domain 5: Outdoor attributes														
Car parking at connected house balcony	1	1	0	1	1	0	1	1	1	1	1	1	10	0.83
Motorcycle parking nearby	1	1	0	1	1	1	1	1	1	1	1	1	11	0.92
Wood stoves outdoor nearby	1	1	1	1	1	1	1	1	1	1	1	1	12	1.00
Domestic garbage burning nearby	1	1	1	1	1	1	1	1	1	1	1	1	12	0.92
Main road nearby	1	1	1	1	1	1	1	1	1	1	1	1	12	1.00
Municipal landfill <500m	1	1	1	1	1	1	1	1	1	1	1	1	12	1.00
Industrial area <500m	1	1	1	1	1	1	1	1	1	1	1	1	12	1.00
Agricultural area <500m	1	1	1	1	1	1	1	1	1	1	1	1	12	1.00
Mines or quarry <500m	1	1	1	1	1	1	1	1	1	1	1	1	12	1.00
Construction site <500m	1	1	1	1	1	1	1	1	1	1	1	1	12	1.00
Outdoor poultry or pet	1	1	0	1	1	1	1	1	1	1	1	1	11	0.92
													^b^S-FVI/Ave	0.97

Reliability analysis

The pooled kappa agreement of inter-rater reliability analysis for five domains (sociodemographic, family history, medical history, household attributes, and outdoor attributes) was 0.76 (95% CI: 0.70, 0.83; p < 0.001), 0.90 (95% CI: 0.79, 0.99; p < 0.001), 0.88 (95% CI: 0.77, 0.99; p < 0.001), 0.81 (95% CI: 0.69, 0.92; p < 0.001), and 0.82 (95% CI: 0.71, 0.94; p < 0.001). The average kappa value for the combined domains was 0.88 (95% CI: 0.81-0.95; p<0.001), and the value indicated good reliability in assessing all domains of the checklist. According to Landis et al., a kappa agreement value of more than 0.80 indicates almost perfect reliability [30].

Final validated checklist

The final validated checklist was called the validated HAPBAC-Checklist; it consisted of five domains and 59 items: sociodemographic (three subdomains, 17 items), family history (three items), medical history (7 items), household attributes (21 items), and outdoor attributes (11 items).

## Discussion

Ensuring optimal household air quality is vital for children’s health and overall well-being. Unfortunately, the evaluation of household air quality remains inadequate, as a comprehensive checklist has not yet been established in Malaysia. Therefore, the development of a new household air quality checklist in the Malay language is essential for widespread adoption, especially among local healthcare authorities and professionals. The household air quality checklist developed in the present study aims to encompass all attributes relevant to household air pollution.

The initial phase involved a comprehensive review of existing tools, checklists, and research. This was complemented by interviews with parents to ensure that the checklist incorporates up-to-date, relevant information, making it suitable for the local context. The content validation process involved a panel of six experts, in accordance with recommendations to have at least six and as many as 10 people [23,24]. Content validation is key to showing the relevance of the constructed items when accessing the respective domains.

Any newly developed checklist or questionnaire has to be assessed for face validity to determine whether it is appropriate and feasible for use by the target population. During this study, raters evaluated the clarity of instructions, the language employed in the assessment list, and overall comprehensibility, following the guidelines proposed by Yusoff [26]. Their evaluation found satisfactory clarity and comprehensibility. Cohen’s kappa agreement was employed to assess the reliability of the checklist. This statistical measure quantifies the agreement between two raters. A pooled kappa estimator was utilized for each domain, which provides a better precision of estimates compared to the averaged method [31]. In this study, the results were favorable, indicating that the checklist is reliable for field use. However, it is important to note that Cohen’s kappa agreement does not directly reveal the accuracy of either rater’s observations [29]. Cohen’s kappa agreement was deemed adequate for reliability analysis, considering that the checklist was designed to measure a single construct, and all its items were directly related to that construct. Furthermore, the checklist items were binary, with minimal variance in responses [32]. 

Initially, the HAPBAC-Checklist was designed to address household air pollution originating from indoor sources. However, recognizing the impact of outdoor factors is crucial, as a multitude of outdoor air pollutants find their way indoors through pathways such as infiltration, ventilation, and human activities [14]. Furthermore, when developing a comprehensive checklist, it is essential to consider children’s backgrounds, including any family history of atopy, and their medical conditions. The checklist was developed with due consideration of the local lifestyle and consequent household exposure in Kota Bharu, Kelantan, Malaysia. It encompassed common factors related to childhood BA development at the household level. However, certain exposures, such as emissions from printers or pollen from cotton trees, were not included because they are uncommon. In addition, the checklist did not count exposures beyond the home, such as those encountered in schools or frequently visited areas such as playgrounds or malls. While the checklist may have limitations when applied to other regions due to cultural and environmental differences, it is likely relevant in areas that share similar exposures, especially within Malaysia and certain other parts of Southeast Asia.

The final HAPBAC-Checklist has been validated and is reliable for use by primary healthcare providers to assess the risk of household air pollution in the development of childhood BA. Additionally, the checklist can serve as a valuable tool for local municipalities during routine inspections and preventive activities related to respiratory diseases. Furthermore, widespread use of the checklist will significantly enhance awareness of allergen and factor identification among the community and local authorities regarding respiratory health.

## Conclusions

This newly developed HAPBAC-Checklist is valid and reliable for evaluating household air pollution with respect to the risk of childhood bronchial asthma onset. An acceptable number of expert panels assessed the checklist for content and face validity, and reliability analysis was performed by examining Cohen’s kappa agreement. The final checklist comprises five domains with 59 items and is recommended for health sectors, local authorities, and non-governmental organizations, who can derive benefits by using the checklist as an integral component of health promotion programs and house regulations. For future studies, the checklist can serve as a foundation for larger-scale research. This approach will enhance the checklist by identifying additional factors based on local exposures.

## Appendices

**Table 5:** The HAPBAC-Checklist in English translation Note: The checklist was originally written in Malay. The English translation provided here serves the purpose of facilitating understanding.

HAPBAC-Checklist (HAPBAC = Household Air Pollution attributes for Bronchial Asthma in Children)															
Version: 9/12/2023															
Checklist objective: The checklist is intended to be used by researchers and trained personnel to record visual observations from the household environment and interview parents or guardians in Malaysian households. Note: The checklist was originally written in Malay. The English translation provided here serves the purpose of facilitating understanding															
User instruction:	The method of obtaining checklist information is through interviews with mothers, fathers, or valid guardians, AND through observing the children’s residences Fill in the blanks or mark ✔ in the relevant boxes														
Staff / User Code	: ________________________________________														
Household Code	: ________________________________________														
Respondent	: ________________________________________ (Mother, father or valid guardians only)														
Domain 1	Sociodemographic														
a) Child background															
1. Age	: _____ Year								Date of birth: ________________						
2. Sex	▢ Male or ▢ Female														
3. Race	▢ Malay ▢ Chinese ▢ Indian ▢ Others; please specify: ____________________________________														
4. School name	: ______________________________________________________________________														
5. School session	▢ Morning or ▢ Afternoon/evening														
6. Extra school session	▢ Yes or ▢ No If Yes, please state the time started: __________ ended:____________														
b) Parents (or guardian) information															
7. Father’s occupation	: _____________________________ State dash (-) if no information														
8. Mother’s occupation	: _____________________________ State dash (-) if no information														
If the child not being cared for by the father or the mother, please specify the guardian’s occupation : _____________________________															
As for education level below, please state the highest education level according to the code below: 0 – Not schooling / Informal 1 – Primary school 2 – Secondary school / SPM certificate 3 – Tertiary certificate / Diploma 4 – Bachelor’s degree or higher State dash (-) if no information															
9. Father’s education	▢							10. Mother’s education							▢
If the child not being cared for by the father or the mother, please specify the guardian’s education ▢															
11. Household income	RM ____________________________ per month														
c) Household information															
12. House location	_________________________________________________________ Simplified address: Compound / sub district area Postcode: ______________________														
13. Duration of stay (at current house)	________ years														
14. House type	▢ Bungalow ▢ Lot low cost house ▢ Semi-detached ▢ Terrace ▢ Townhouse ▢ Apartment or condominium ▢ Low cost high rise / Flat ▢ Others, please specify: ______________														
15. House main structure	▢ Concrete ▢ Wood ▢ Mixed wood & concrete ▢ Others, please specify: ______________														
16. No. of bedrooms	: __________________							17. No. of occupants						: ______________ people(s)	
Domain 2	Family Allergy History														
1. Biological father’s history	You may tick more than one: ▢ No history ▢ Asthma ▢ Allergy rhinitis ▢ Eczema ▢ Others allergy (ie. food, medication or others) ▢ No information available														
2. Biological mother’s history	You may tick more than one: ▢ No history ▢ Asthma ▢ Allergy rhinitis ▢ Eczema ▢ Others allergy (ie. food, medication or others) ▢ No information available														
3. Biological sibling’s history	You may tick more than one: ▢ No history ▢ Asthma ▢ Allergy rhinitis ▢ Eczema ▢ Others allergy (ie. food, medication or others) ▢ No information available														
Domain 3	Child medical History														
1. Gestational age at child’s birth	▢ At term ▢ Pre-term (<37 weeks) ▢ Not sure														
2. Maternal smoking during pregnancy	▢ Yes ▢ No														
3. Breast feeding history for the first six month	▢ Exclusive breast feeding ▢ Not exclusive (mixed) ▢ Formula milk only														
4. Congenital or chronic illness	▢ Yes ▢ No If Yes, please specify: _______________________________________														
5. History of hospital admission due to respiratory infection?	▢ Yes ▢ No If Yes, please specify: _______________________________________														
6. Has the child been diagnosed with asthma?	▢ Yes ▢ No If Yes, please specify year/month of diagnosis made: _______________														
7. Any other medical condition besides asthma?	▢ Yes ▢ No If Yes, please specify: _______________________________________														
Domain 4	Household Attributes														
1. Any parents or guardian smoking?										▢ Yes ▢ No					
2. Any house occupant (including parents or guardian) in the house currently smoking?										▢ Yes ▢ No					
3. Any house occupant (including parents or guarding) in the house currently vaping?										▢ Yes ▢ No					
4. Is visible cigarette but or an ashtray seen in the house or surrounding structure?										▢ Yes ▢ No					
5. Any pet(s) in the house?			▢ Yes ▢ No If Yes, please state type of pet(s): ▢ Cat ▢ Dog ▢ Bird/Chicken ▢ Rabbit/Rodents ▢ Others, please specify:_________												
6. Carpet(s) in the house?			▢ Yes ▢ No												
7. Sign(s) of mould in the house			▢ Yes ▢ No. If Yes, please state, which part:_____________________												
8. Indoor use of aerosol/spray pesticide(s) at least once a week?												▢ Yes ▢ No			
9. Indoor use of mosquito coil at least once a week?												▢ Yes ▢ No			
10. Acquired any new wood furniture(s) in the past 12 months?												▢ Yes ▢ No			
11. Any part of the house been newly painted in the past 12 months?												▢ Yes ▢ No			
12. Use of air conditioning while sleeping almost every night (≥ 4 nights) in a week?												▢ Yes ▢ No			
13. House cleaning frequency				▢ Everyday ▢ Not every day but at least once a week ▢ Not every week but at least once a month ▢ Less than once a month/ once in a few months											
14. House cleaning (main) method				▢ Vacuum cleaner ▢ Sweeping											
15. Indoor use of aerosol/spray cleaning product(s)?												▢ Yes ▢ No			
16. Kitchen has a window(s)?					▢ Yes ▢ No If Yes, the window(s) keep open while cooking? ▢ Yes ▢ No										
17. Kitchen has ventilation fan(s)?					▢ Yes ▢ No If Yes, the fan(s) operating while cooking? ▢ Yes ▢ No										
18. Fried cooking method of the days (≥ 4 days) in a week?													▢ Yes ▢ No		
19. Use of wood stove or any form of biomass fuel stove in the house?					▢ Yes ▢ No If Yes, please specify the usage frequency: ▢ Everyday ▢ Not every day but at least once a week ▢ Not every week but at least once a month ▢ Less than once a month/ once in a few months										
20. Indoor fragrance use?					▢ Yes ▢ No If Yes, please specify the type: ▢ Incense / burning ▢ Aerosol / spray ▢ Others, please specify: ________________________										
21. HEPA filter device used most of the days in a week? *HEPA = High Efficiency Particulate Air					▢ Yes ▢ No										
Domain 5		Outdoor Attributes													
1. Any motorcar (including any vehicles (≥ 4 tyres) parked in the balcony or structure directly connected to the house?										▢ Yes ▢ No					
2. Any motorcycle parked in the structure directly connected to the house?										▢ Yes ▢ No					
3. Use of wood stove or any form of biomass fuel stove in the house area (including adjacent neighbour’s house)							▢ Yes ▢ No If Yes, please specify the usage frequency: ▢ Everyday ▢ Not every day but at least once a week ▢ Not every week but at least once a month ▢ Less than once a month/ once in a few months								
4. Domestic garbage burning in the house area (including adjacent neighbour’s house)							▢ Yes ▢ No If Yes, please specify the usage frequency: ▢ Everyday ▢ Not every day but at least once a week ▢ Not every week but at least once a month ▢ Less than once a month/ once in a few months								
5. House radius to the nearby main road? User can utilise a map application to find the distance											: _____________ meter				
6. Nearby (<500 meter radius) landfill											▢ Yes ▢ No				
7. Nearby (<500 meter radius) industrial area											▢ Yes ▢ No				
8. Nearby (<500 meter radius) agricultural area											▢ Yes ▢ No				
9. Nearby (<500 meter radius) quarry or mine area											▢ Yes ▢ No				
10. Nearby (<500 meter radius) construction area											▢ Yes ▢ No				
11. Any pet(s) or animal in the house area? This includes animal(s) that are not owned by the house occupants but frequently visit to the house area, or animal(s) in the adjacent neighbour’s house that produce unpleasant smells						▢ Yes ▢ No If Yes, please state type of animal(s): ▢ Cat ▢ Dog ▢ Bird ▢ Chiken/Duck ▢ Cow/Goat/Buffalo ▢Horse ▢ Others, please specify: _______________________									
